# Mannan oligosaccharides trigger multiple defence responses in rice and tobacco as a novel danger‐associated molecular pattern

**DOI:** 10.1111/mpp.12811

**Published:** 2019-05-16

**Authors:** Haoyu Zang, Shanshan Xie, Bichun Zhu, Xue Yang, Chunyan Gu, Benjin Hu, Tongchun Gao, Yu Chen, Xuewen Gao

**Affiliations:** ^1^ College of Plant Protection Nanjing Agricultural University, Key Laboratory of Integrated Management of Crop Diseases and Pests, Ministry of Education Nanjing 210095 PR China; ^2^ Institute of Plant Protection and Agro‐Products Safety Anhui Academy of Agricultural Sciences Hefei 230031 China; ^3^ The National Key Engineering Lab of Crop Stress Resistance Breeding, College of Life Sciences Anhui Agricultural University Hefei 230036 China

**Keywords:** *Bacillus*, danger-associated molecular pattern, defence response, degree of polymerization, mannan oligosaccharides, rice and tobacco

## Abstract

Oligosaccharide, a typical danger‐associated molecular pattern (DAMP), has been studied and applied as plant defence elicitor for several years. Here, we report a novel oligosaccharide, mannan oligosaccharide (MOS) with a degree of polymerization of 2–6, which was hydrolysed from locust bean gum by a newly reported enzyme, BpMan5. The MOS treatment can significantly enhance the generation of signalling molecules such as intracellular Ca^2+^ and reactive oxygen species. Subsequent defence events like stomata closure and cell death were also caused by MOS, eventually leading to the prevention of pathogen invasion or expansion. Transcriptional expression assay indicated that MOS activated mitogen‐activated protein kinase cascades in tobacco and rice via different cascading pathways. The expression levels of the defence‐related genes *PR‐1a* and *LOX* were both up‐regulated after MOS treatment, suggesting that MOS may simultaneously activate salicylic acid and jasmonic acid‐dependent signalling pathways. Furthermore, liquid chromatography‐mass spectrometry analysis showed that MOS led to the accumulation of four phytoalexins (momilactone A, phytocassane A, phytocassane D, and phytocassane E) in rice seedling leaves within 12–24 h. Finally, MOS conferred resistance in rice and tobacco against *Xanthomonas oryzae* and *Phytophthora nicotianae*, respectively. Taken together, our results indicated that MOS, a novel DAMP, could trigger multiple defence responses to prime plant resistance and has a great potential as plant defence elicitor for the management of plant disease.

## Introduction

Unlike vertebrate animals, plants rely entirely on an innate immune system for their resistance against various pathogens. Plant innate immune response has two layers: pattern‐triggered immunity (PTI) and effector‐triggered immunity (ETI) (Chisholm *et al.*, 2006; Jones and Dangl, 2006). The first layer immune response is initiated by recognition of microbe or modified plant‐derived molecules such as flagellin, lipopolysaccharides, chitin, and oligogalacturonides (OGAs) through pattern‐recognition receptors (PRRs) located on the cell membrane (Hayafune *et al.*, 2014; Zipfel *et al.*, 2004). All of these molecules, which could universally be described as ‘patterns that elicit immunity’ (PEIs), can be described as chemical elicitors, microbe‐associated molecular patterns (MAMPs), pathogen‐associated molecular patterns (PAMPs), and/or danger‐associated molecular patterns (DAMPs, also known as ‘classic’ damage‐associated molecular patterns) (Mazzotta and Kemmerling, 2011; Wiesel *et al.*, 2014). Perception of PTI leading to plant resistance. Some pathogens have evolved to successfully suppress PTI by secreting effectors into host cells, resulting in effector‐triggered susceptibility (ETS) (Zipfel, 2014). In turn, plants have also evolved resistance proteins to recognize effectors, resulting in ETI (Elmore *et al.*, 2011). During the plant–microbe interaction process, not only can cell wall degrading enzymes secreted by microbe themselves be recognized as MAMPs/PAMPs by PRRs, but also their enzymatic products can function as general elicitors to trigger plant defence responses (Poinssot *et al.*, 2003; Shibuya and Minami, 2001). In fact, both endogenous DAMPs and microbe‐derived PAMPs are considered to be danger signals (Gust *et al.*, 2017). Among these, oligosaccharide derived from plant cell walls is one of the most typical representative elicitors. Hence, degradation products from natural polysaccharides such as plant cell walls can also be termed DAMPs or microbe‐induced molecular patterns (MIMPs) (Ferrari *et al*., 2013, Mackey and Mcfall, 2006).

Oligosaccharides are low molecular weight saccharide polymers with degrees of polymerization (DP) between 2 and 10, mainly derived from plant cellulose or hemicellulose like pectins, xyloglucans, cellodextrins etc. (Aziz *et al.*, 2007; Laere *et al.*, 2000; Paulert *et al.*, 2010), or from fungal cell walls or the exoskeleton of arthropods such as chitin, chitosan, and β‐glucan (Aziz *et al.*, 2003; Li *et al.*, 2009; Yin *et al.*, 2010). Various kinds of oligosaccharides, such as chitosan oligosaccharides (COSs), OGAs, and xyloglucan oligosaccharides (XGOs), have been confirmed to participate in plant growth and defence responses (Benedetti *et al.*, 2015; Kaida *et al.*, 2010). For example, chitin oligosaccharides derived from chitin can be recognized by the plasma membrane glycoprotein CEBiP containing two extracellular LysM motifs (Shimizu *et al.*, 2010). COS, an analogue of chitin oligosaccharides, is also effective in eliciting plant innate immunity against plant diseases. Once COS is detected, the signalling transmits quickly and induces early defence responses, including the release of Ca^2+^ into the cytosol, mitogen‐activated protein kinase (MAPK) activation, reactive oxygen species (ROS) generation, hypersensitive responses activation, and accumulation of abscisic acid, jasmonates, phytoalexins, and PR‐proteins (Yin *et al.*, 2010). OGAs with a DP ranging from 10 to 16, released from the main component of plant cell wall pectin, are well‐characterized DAMPs. OGA can be sensed by two cell wall‐associated receptor‐like kinases WAK1 and WAK2, then triggers plant immunity via activation of MAPKs, an oxidative burst, and accumulation of pathogenesis‐related proteins, and finally results in enhanced resistance against pathogen invasion (Brutus *et al.*, 2010; Denoux *et al.*, 2008; Galletti *et al.*, 2011). The elicitor activities proved to be associated with the sugar composition, structure, DP, and DA of oligosaccharides (Darvill *et al.*, 1992; Yin *et al.*, 2016). Many studies have confirmed some important roles of oligosaccharides in activating plant innate immunity, whereas the DP of these oligosaccharide elicitors was mostly greater than 7 (Aziz *et al.*, 2004; Yin *et al.*, 2016). Relatively fewer studies have focused on the function of oligosaccharides with lower DPs. It still remains unclear whether mannan oligosaccharides with DP less than 7 can still function as DAMPs and activate the plant defence response.

*Bacillus* spp., a typical plant growth promoting rhizobacteria, can promote plant growth by improving the availability of nutrients, producing phytostimulators like indole‐3‐acetic acid (IAA), and protecting plants against various pathogen infections by activating plant innate immunity (Lugtenberg and Kamilova, 2009). Some secondary metabolites produced by *Bacillus* contribute to the process of activating plant innate immunity. For instance, purified surfactins and fengycins, a kind of cyclic lipopeptide widely produced by *Bacillus*, induce plant immunity to protect tomato against *Botrytis cinerea* infection (Ongena *et al.*, 2007). The volatile organic compound 2,3‐butanediol released from *Bacillus subtilis* GB03 also enhances *Arabidopsis* resistance against *Pectobacterium carotovorum* (Ryu *et al.*, 2004). Furthermore, *Bacillus amyloliquefaciens* FZB42 suppressed miR846 expression to induce *Arabidopsis* innate immunity via a JA‐dependent signalling pathway (Xie *et al.*, 2018). These studies confirmed the functional mechanism of *Bacillus* in activating plant innate immunity. Nevertheless, *Bacillus* spp. also encode a series cellulase or hemicellulase regarding plant cell well degradation (Maki *et al.*, 2009). It is a fascinating issue to explore whether the degradation products, i.e. different kinds of oligosaccharides, can be considered an additional approach to trigger plant resistance.

In our previous study, we identified a novel thermostable mannanase BpMan5, which was encoded by *Bacillus pumilus* GBSW19 (Zang *et al.*, 2015). By using BpMan5, a novel oligosaccharide, mannan oligosaccharides (MOS) with a DP of 2–6 can be hydrolysed from locust bean gum. Here, we investigate the intracellular Ca^2+^ and ROS content after MOS treatment. Typical defence responses like stomata closure and hypersensitive responses in *Nicotiana benthamiana* and *Oryza sativa* were also examined. In addition, the defence‐related gene expression levels as well as phytoalexin contents were both quantified in response to MOS. Furthermore, the protective effects of these elicitors in rice and tobacco against *X. oryzae* and *P. nicotianae*, respectively, were also examined.

## Results

### MOS with a DP of 2–6 can be hydrolysed from LBG using BpMan5

**Figure 1:**
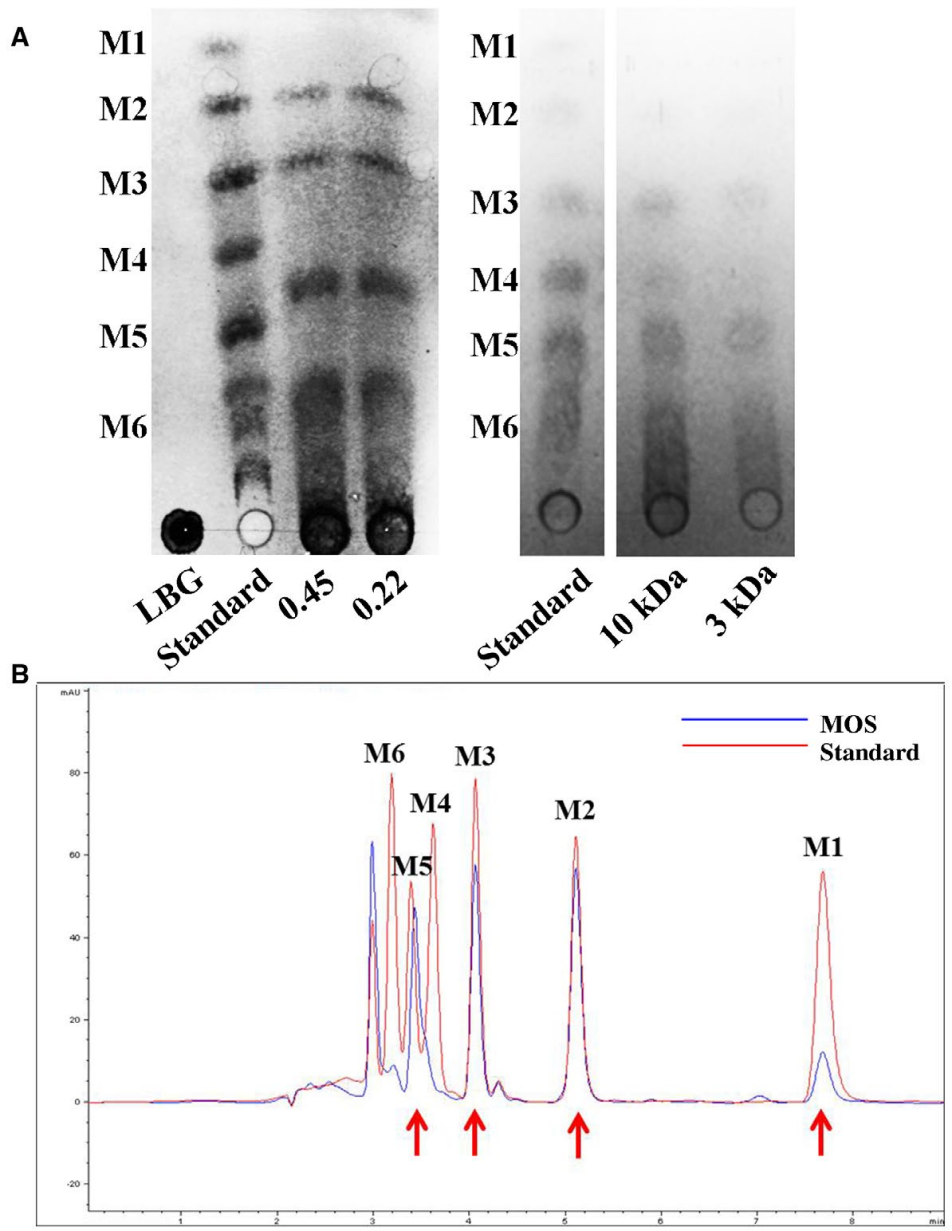
Purification (A) and sugar composition analysis (B) of MOS. (A) TLC showed that polysaccharide‐free hydrolysis products were obtained through centrifugation‐microfiltration‐ultrafiltration steps. Purified BpMan5 was incubated with 10 mg/mL LBG for 24 h at 50 °C. The hydrolysis products were purified successively through centrifugation, microfiltration, and ultrafiltration, and finally detected using TLC. Mobile phase: mixed liquor of ethyl acetate, acetic acid, ethanol, and water (12:3:3:1, v/v). M1, mannose; M2, mannobiose; M3, mannotriose; M4, mannotetrose; M5, mannopentaose; M6, mannohexaose; LBG, locust bean gum; standard: a standard mixture of mannose (M1) to mannohexaose (M6); 0.45, microfiltration using 0.45 μm microfiltration filter membrane; 0.22, microfiltration using 0.22 μm microfiltration filter membrane; 10 kDa, ultrafiltration using 10 kDa tubular ultrafiltration; 3 kDa, ultrafiltration using 3 kDa tubular ultrafiltration. The red circle indicates that the polysaccharides can be ultimately removed through 3 kDa tubular ultrafiltration. (B) HPLC analysis confirmed that the MOS mixture was mainly composed of mannobiose, mannotriose, and mannopentaose. MOS mixtures were first labelled with PMP and then loaded into an Agilent 1200 series LC system equipped with a C18 reverse column. Mobile phase: 0.1 M NH_4_OAc and acetonitrile at a ratio of 78:22. Flow rate: 1 mL/min. Standard: a standard mixture of mannose (M1) to mannohexaose (M6).

In our previous study, a thermal‐stable, ion‐activated hemicellulose, BpMan5, was cloned from the biocontrol agent *Bacillus pumilus* GBSW19. Bioconversion of natural polymer such as cheap locust bean gum (LBG) into high value‐added oligosaccharides (MOS) can be achieved with BpMan5 (Zang *et al.*, 2015). Based on these findings, we developed the optimum conditions for MOS preparation from LBG. However, high polysaccharide content can still be found in enzymatic hydrolysates. Thus, we adopt a series of steps, including centrifugation, microfiltration (0.22 μm), and ultrafiltration (3 kDa), to finally obtain a polysaccharide‐free oligosaccharides mixture, which was confirmed by thin‐layer chromatography (TLC) and high‐performance liquid chromatography (HPLC) analysis (Fig. 1). HPLC analysis also revealed that MOS generated with a DP of 2–6 were mainly M2, M3, and M5 (Fig. 1B).

### MOS with a DP of 2–6 leads to Ca^2+^ influx and ROS generation in guard cells of *N. benthamiana*

**Figure 2:**
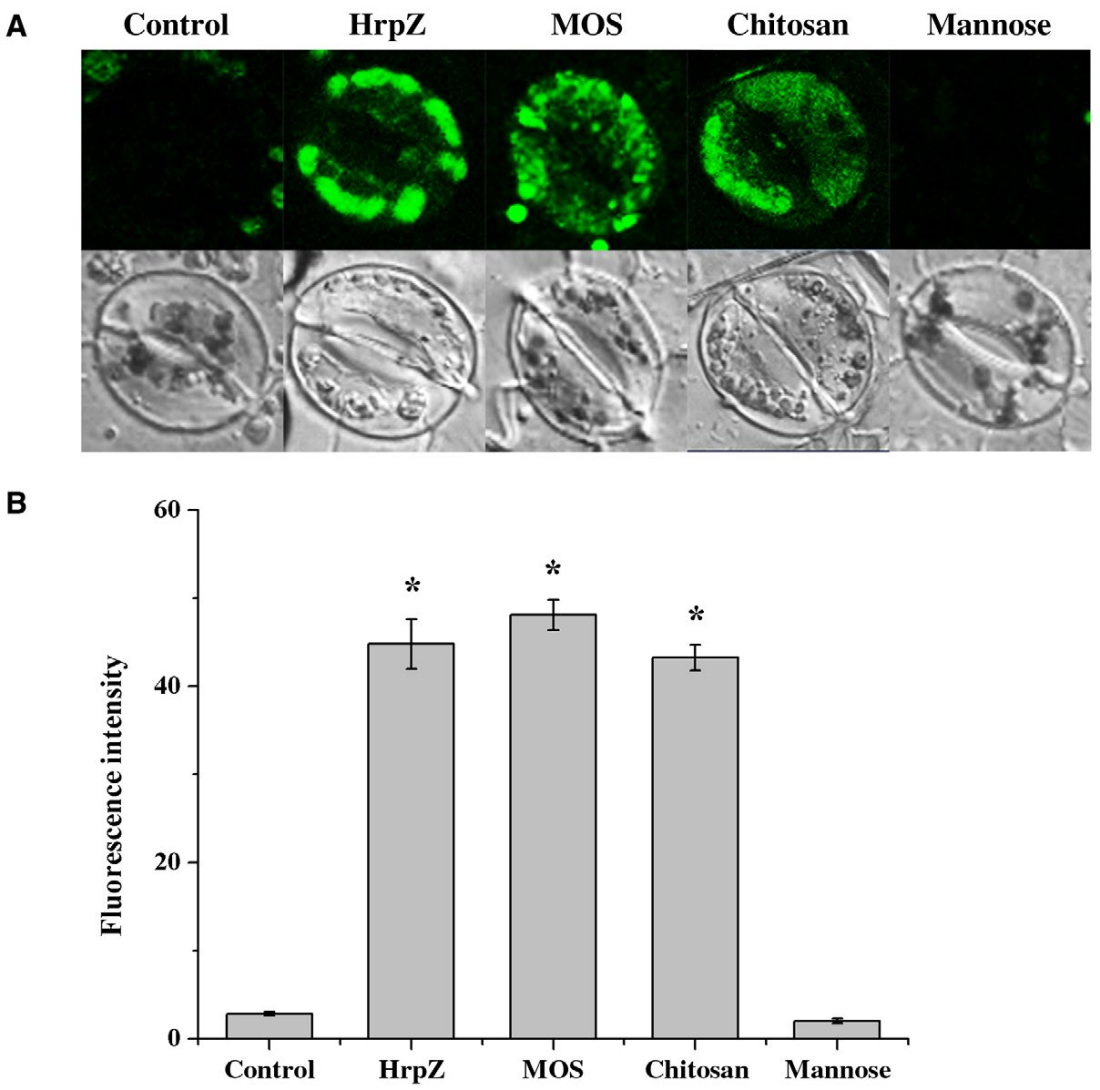
MOS promotes intracellular Ca^2+^ accumulation in the guard cells of *N. benthamiana*. Calcium‐specific fluorescence probe fluo‐3 AM was pre‐incubated with epidermal peels at 4 °C, then kept at room temperature for 1 h. The fluorescence was observed by laser confocal microscope after incubation with control buffer, HrpZ (500 μg/mL), chitosan (1000 μg/mL), mannose (13 μg/mL), and MOS (200 μg/mL) for 3 h. (A) Representative images (enlarged image). (B) Quantitative analysis of Ca^2+^ concentration. Results are presented as the average fluorescence intensity of guard cells using ZEN software. The experiments were repeated three times. * indicates significant differences according to Fisher’s least‐significant difference test (*P* < 0.05) using SPSS software (*n* = 50).

In plant cells, the cytoplasmic Ca^2+^ usually stays at low concentration, whereas certain biotic stresses, such as pathogen infection and elicitor, promote Ca^2+^ influx from extracellular to cytoplasm, thus leading to a rapid transient cytoplasmic Ca^2+ ^increase. Ca^2+^ functions as an important secondary messenger to trigger the defence response. To investigate whether MOS with a DP of 2–6 leads to Ca^2+^ influx, the cytoplasmic Ca^2+^ levels were evaluated by fluorescent‐labelled Fluo‐3AM. Harpin protein HrpZ_S1_ and chitosan were chosen as positive controls (Iriti and Varoni, 2015; Zhang *et al.*, 2009). No obvious fluorescence was detected in mannose or control‐treated guard cells, whereas significant fluorescence was shown in MOS, chitosan, and HrpZ_S1_‐treated guard cells (Fig. 2A). The quantification of fluorescence intensity showed a similar result (Fig. 2B), indicating that MOS promotes Ca^2+ ^influx in guard cells.

**Figure 3:**
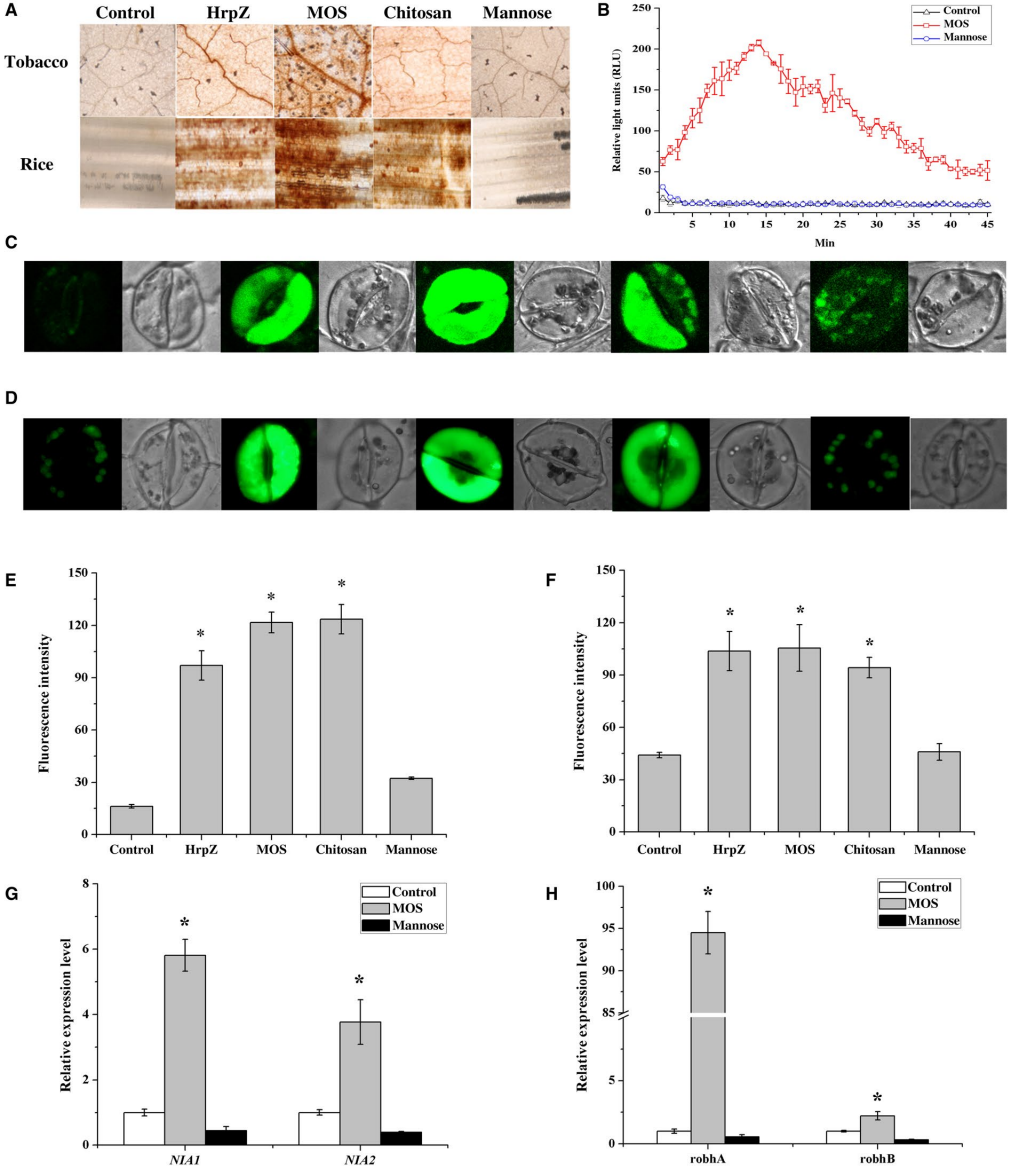
MOS enhances plant H_2_O_2_ (A, B), NO (C, E, G), and ROS (D, F, H) generation. (A) Tobacco and rice leaves were injected with control buffer, HrpZ (500 μg/mL), chitosan (1000 μg/mL), mannose (13 μg/mL) or MOS (200 μg/mL) for 6 h, then cut off and soaked in DAB dye liquor for 10 h. The leaves were bleached in boiling ethanol and then visualized under a microscope. (B) H_2_O_2_ production was measured in *N. benthamiana* with luminol‐based assay after treatments with MOS (200 μg/mL), mannose, and control. Results are average ± SD (*n* = 4). NO‐sensitive dye DAF‐2DA and ROS dye DHR were loaded into cells of the epidermal peels, and fluorescence was measured after incubation with control buffer, HrpZ (500 μg/mL), chitosan (1000 μg/mL), mannose (13 μg/mL), and MOS (200 μg/mL). Representative images are shown in (C) for NO generation and (D) for ROS generation. (E, F) Quantitative analysis of NO and ROS generation using DAF‐2DA or DHR. Results are presented as the average fluorescence intensity of guard cells using ZEN software. (G, H) Transcript levels of marker genes associated with NO and ROS accumulation. Tobacco leaves were harvested after 3 h treatment with MOS, mannose, and double distilled water (ddW). *NIA1,* NIA2, *NbrobhA*, and *NbrobhB* were quantified by qRT‐PCR. Values represent means ± SE. The experiments were repeated three times. * indicates significant differences according to Fisher’s least‐significant difference test (*P* < 0.05) using SPSS software.

ROS including hydrogen peroxide (H_2_O_2_) and nitrogen oxide (NO) act as signalling molecules and participate in the plant defence response against pathogen. To investigate whether MOS with a DP of 2–6 affects ROS accumulation, we first determined the amount of H_2_O_2_ using the DAB staining method. A large amount of brown deposits were observed on the leaves of *N. benthamiana* and *O. sativa* after treatment with MOS, chitosan, and HrpZ_S1_, indicating that MOS induced H_2_O_2 _accumulation (Fig. 3A). Furthermore, a time‐course ROS production assay of tobacco leaves in response to MOS treatment was performed based on luminescence detection (Bellincampi *et al.*, 2000; Gigli‐Bisceglia *et al.*, 2015). Figure 3B shows that H_2_O_2_ was detected approximately 2 min after MOS application and production reached a maximum after the next 12 min. Thereafter, H_2_O_2_ concentration declined back to the initial level after 40 min. In addition, NO and ROS levels were monitored using fluorescent dyes DAF‐2DA and rhodamine 123, and the expression levels of NO‐ and ROS‐related genes were also detected. The results show that MOS significantly induced the fluorescent intensities in guard cells compared to the control (Fig. 3C–F). Moreover, the expression levels of genes associated with NO (*NIA1*, *NIA2*) and ROS (*robhA*, *robhB*) accumulation were both induced (Fig. 3G‐H). These results indicate that MOS with a DP of 2–6 can lead to ROS accumulation. It should be noted that mannose treatment also induced a slight enhancement of NO production compared to the control. This might be due to the report that mannose also has an effect on ROS production in a dose‐ and time‐dependent manner (Li *et al.*, 2016). However, the NIA1/2 were not changed compared to the control, indicating that the mannose affects the synthesis of nitric oxide through other pathways (Crawford, 2005).

### MOS with a DP of 2–6 activates MAPK cascades and defence‐related gene transcription, and enhances accumulation of four phytoalexins

**Figure 4:**
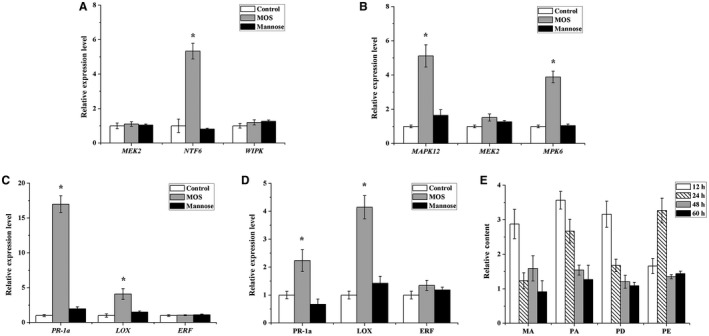
MOS induced the expression levels of MAPK genes and defence‐related genes in *N. benthamiana* (A, C) and *O. sativa* (B, D) and the phytoalexin accumulation in *O. sativa* (E). (A, B) Tobacco and rice were sprayed with MOS (200 μg/mL), mannose, and control. Six leaves per sample were collected after 3 h. Expression levels of MAPK genes were detected using qPCR. (C) Upper leaves of tobacco seedlings were collected 12 h after foliage application of 200 mg/L MOS on lower leaves, and transcriptional levels of defence‐related genes were performed. (D, E) Rice leaves were cut off 12 h after root irrigation with 200 mg/L MOS, and transcriptional levels of defence‐related genes were performed. Phytoalexin levels in rice were detected using LC‐MS after root‐irrigation with MOS for different periods (0,12, 24, 48, and 60 h). Seedlings soaked in sterile water were used as a control. Phytoalexin levels in rice treated with MOS for different periods were compared with that at 0 h. MA, momilactone A; PA, phytocassane A; PD, phytocassane D; PE, phytocassane E. Values represent means ± SE.

The MAPK cascade is highly conversed in eukaryotes and can be activated by both biotic and abiotic signals. In plants, MAPK can be activated by PRRs to participate in multiple plant defence responses (Meng and Zhang, 2013). According to real‐time PCR results, MOS treatment enhanced the expression level of *NFT6* in tobacco (Fig. 4A), whereas in rice plants the *MAPK12* and *MPK6* were up‐regulated (Fig. 4B). These results indicate that MOS with a DP of 2–6 indeed activates the MAPK cascade pathways in plants, but on the other hand there might be differences in activation patterns among different plants. To investigate whether MOS with a DP of 2–6 subsequently activated plant systemic resistance or not, we monitored the expression levels of defence‐related genes in dicotyledon *N. benthamiana* and monocotyledon *O. sativa*. Salicylic acid (SA), jasmonic acid (JA), and ethylene (ETH) play important roles in activating the defence response against pathogen infection. *PR‐1a* (encoding acidic pathogenesis‐related protein), *LOX* (involved in JA synthesis) and *ERF1* (ETH responsive gene) are the marker genes of the SA‐, JA‐ and ETH‐dependent defence signalling pathways, respectively (Mauch‐Mani *et al.*, 2017). The real‐time PCR assay showed induced expression levels of *PR‐1a* and *LOX* in *N. benthamiana* and *O. sativa* after MOS treatment, while the expression level of *ERF1* remained unchanged (Fig. 4C–D).

To further confirm the priming process, four phytoalexins in rice seedlings were detected using liquid chromatography‐mass spectrometry (LC‐MS). Phytoalexins are typical antimicrobial and antioxidative substances synthesized by host plants to defend pathogen invasion. They are chemically diverse with different types of characteristic particularly plant species and are commonly considered to be associated with plant resistance. Rice produces a wide array of phytoalexins, such as momilactones, phytocassanes, oryzalexins, and phenolics, in response to pathogen attack (Cho and Lee, 2015). Among these, momilactones and phytocassanes both accumulate on infection by pathogenic fungi *Magnaporthe oryzae* and bacterial *Xanthomonas oryzae* in rice leaves (Hasegawa *et al.*, 2010; Klein *et al.*, 2015). In our study, four kinds of phytoalexins (momilactone A, phytocassane A, phytocassane D, and phytocassane E) in rice were extracted and measured after MOS treatment. Their concentrations all rose to the maximum within 24 h, and then decreased to normal levels at 60 h (Fig. 4E), suggesting that the application of MOS with a DP of 2–6 could improve plant disease resistance by promoting the generation of different phytoalexins.

### MOS with a DP of 2–6 induces hypersensitive responses and stomatal closure in *N. benthamiana* and *O. sativa*

**Figure 5:**
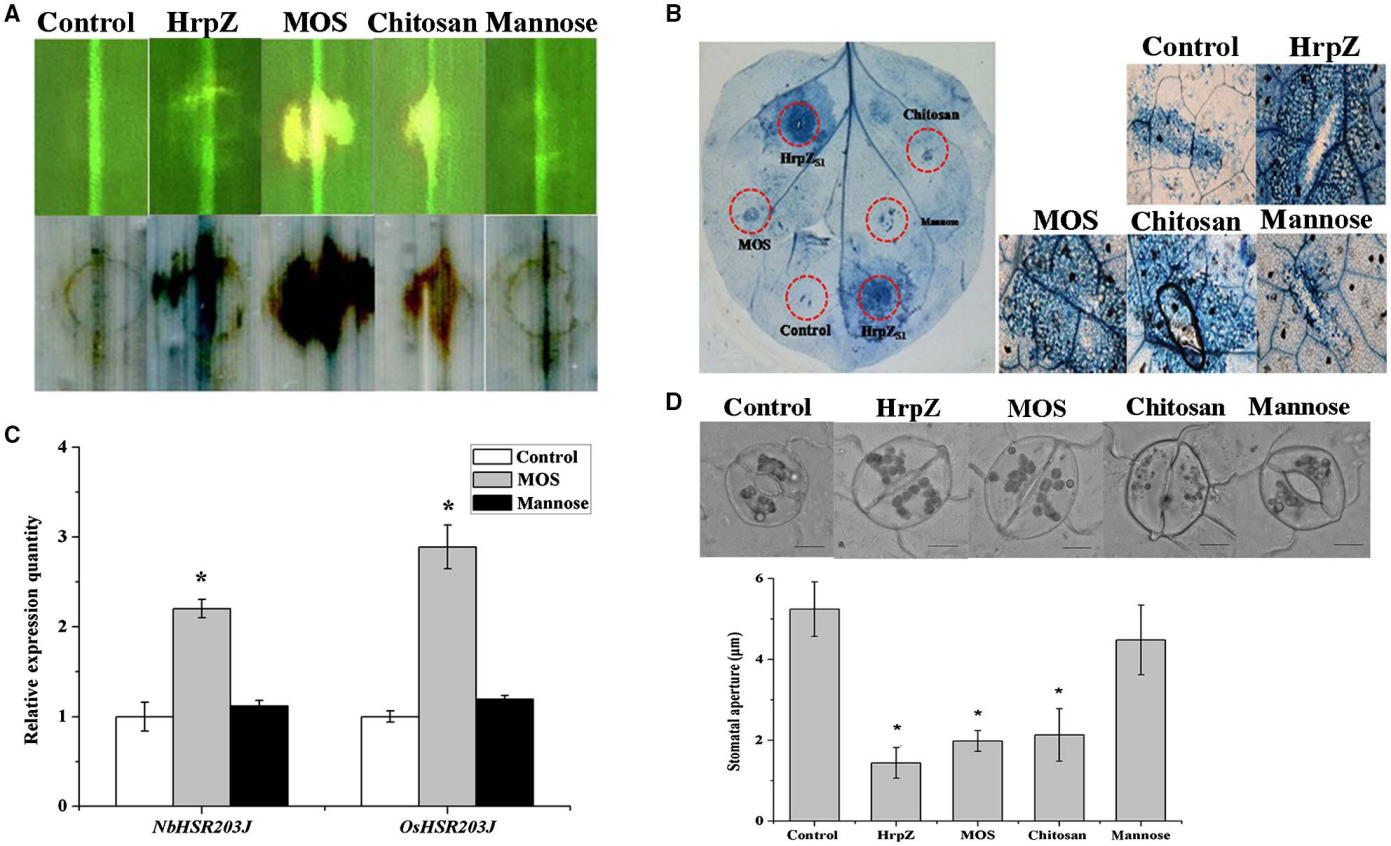
MOS induces hypersensitive responses (A–C) and stoma closure (D) in rice and tobacco. Rice and tobacco leaves were infiltrated with Harpin protein HrpZ_S1 _(500 μg/mL), chitosan (500 μg/mL), MOS (200 μg/mL), mannose (13 μg/mL), and ddW using a 1‐ml syringe without a needle. Injection areas from rice (A) and tobacco (B) were cut down after staining with trypan blue and viewed through a microscope. (C) The transcript levels of HR marker gene HSR203J were quantified by qRT‐PCR. Values represent the means of three replicates. * indicates significant difference compared with the control (*P* < 0.05). The assay was repeated three times. Peeled tobacco epidermis was exposed under light for at least 4 h until the pores were fully open prior to different elicitors or buffer treatment. Pictures were taken after 3 h of incubation with a 40‐fold lens. (D) Stomatal apertures were measured under the same conditions using Olympus CellSense software. * indicates significant difference compared with the control (*P* < 0.05) (*n* = 50). The assay was repeated three times.

To explore whether MOS could induce a typical plant defence response, the hypersensitive responses were assayed after inoculating with MOS, HrpZ_S1_, chitosan, mannose or sterile water. The harpin protein HrpZ_S1 _and chitosan were chosen as positive controls. MOS, chitosan, and HrpZ_S1_ caused necrotic lesions in *O. sativa*, and trypan blue staining analysis showed a consistent result (Fig. 5A). Nevertheless, no obvious necrotic lesions were observed in *N. benthamiana* and death cells could only be detected under a microscope (Fig. 5B). In addition, the expression levels of hypersensitive response marker genes (*OsHSR203J* and *NbHSR203J*) were also induced by MOS treatment (Fig. 5C) (Wendehenne *et al.*, 2002). These results indicate that MOS caused a hypersensitive response in *O. sativa* and a microscopic hypersensitive response in *N. benthamiana*.

Pathogen entry into hosts via stomata, water pores or wounds is an essential step during the infection process. Various elicitors have been confirmed to induce stomatal closure. For example, elicitors (cryptogein and harpin) from microbial pathogens cause hypersensitive response and stomatal closure in tobacco. The effects of MOS on stomatal apertures were investigated according to Chen (Chen *et al.*, 2004). Following 3 h incubation, MOS, chitosan, and HrpZ_S1_ all caused stomatal closure in *N. benthamiana*, and the stomatal apertures were significantly (*P* < 0.05) reduced compared to mannose and control (Fig. 5D). Our results suggest that MOS with a DP of 2–6 induced stomatal closure in plant defence response.

### MOS with a DP of 2–6 enhances *N. benthamiana* resistance against *P. nicotianae* and *O. sativa* resistance against *X. oryzae*

**Figure 6:**
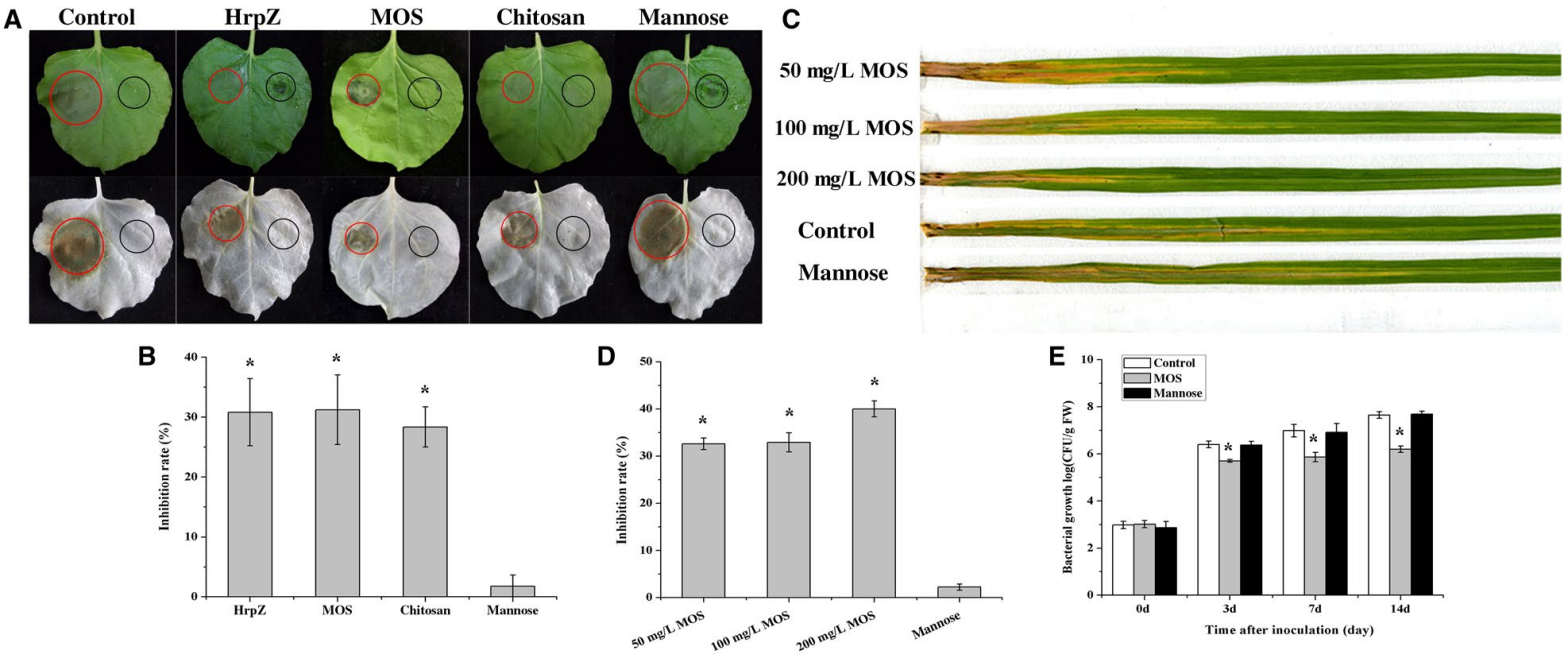
MOS treatment enhance tobacco (A, B) and rice (C–E) resistance against pathogens. (A) The right‐hand sides of 6‐week old tobacco leaves were infiltrated with MOS (200 μg/mL), HrpZ (500 μg/mL), chitosan (1000 μg/mL), mannose (13 μg/mL), and ddH_2_O. The left‐hand sides (red circles) were then inoculated with a 7 × 7 mm hyphal plug of *P. nicotianae*. Disease symptoms were measured after 48 h and then decolorized in ethanol. (B) Resistance evaluation based on the diameters of the lesion spots. Inhibition rate % = (diameter of control － diameter of elicitor)/diameter of control × 100. (C, D) 45‐day‐old rice plants were root‐irrigated with MOS 24 h prior to *Xanthomonas oryze* pv. *Oryzae* inoculation. After 14 days, the lesion lengths were measured. Inhibition rate % = (lesion lengths of control － lesion lengths of elicitor)/lesion lengths of control × 100. € Quantification of *Xoo* growth in rice after root‐irrigation with MOS, mannose, and control. CFU, colony‐forming unit; FW, fresh weight. * indicates significant difference compared with control (*P* < 0.05). Each treatment contains six plants and the experiment was repeated three times.

For further confirmation of the elicitor activity of MOS, *N. benthamiana* resistance against *P. nicotianae* and *O. sativa* resistance against *X. oryzae* were evaluated. The right‐hand sides of 6‐week‐old *N. benthamiana* leaves were infiltrated with MOS, HrpZ_S1_, chitosan, mannose or sterile water, and the left‐hand sides of *N*. *benthamiana* leaves were then inoculated with *P. nicotianae*. Disease lesion sizes were measured and decolorized observation in ethanol. MOS, HrpZ_S1_, and chitosan significantly inhibited *P. nicotianae* extension (Fig. 6A–B). Furthermore, the roots of 30‐day‐old *O. sativa* were inoculated with MOS at different concentrations, and 24 h later *O. sativa* were challenge‐inoculated with *Xanthomonas oryzae* pv. *oryzae* (*Xoo*) by the leaf clipping method. Finally, the lesion lengths were measured after inoculation for 2 weeks. Pretreatment with 50, 100, and 200 mg/L MOS significantly reduced lesion lengths compared to mannose and control, with 200 mg/L MOS working the best of these, reaching control efficiency of 40% (Fig. 6C‐E). All these results suggest that MOS with a DP of 2–6 activatees plant systemic resistance and could be regarded as a novel DAMP.

## Discussion

Numerous studies of oligosaccharide elicitors have been reported since the 1960s. However, these studies are mainly on chitin oligosaccharides and hepta‐β‐glucoside derived from microbial cell walls, and XGOs, β‐1,3‐glucooligosaccharides, and OGA derived from plants. Fewer studies have focused on the elicitor activities of MOS on plant immunity, even though it was already known as an immunosaccharide in aquaculture (Song *et al.*, 2014).

In this study, we introduced a simple and viable method to prepare and preliminarily purify the MOS originating from plant polysaccharide LBG. Using this method, we can convert naturally abundant, easily available material into water‐soluble and polysaccharide‐free oligosaccharides. It is worth pointing out that the MOS we obtained is completely different from previous oligosaccharide elicitors in terms of both monosaccharide composition and degree of polymerization (Aziz *et al.*, 2003, 2007; Laere *et al.*, 2000; Li *et al.*, 2009; Paulert *et al.*, 2010; Yin *et al.*, 2010). Through a series of studies, we proved that MOS triggers various defence and resistance responses in both tobacco and rice, including elevation of intracellular Ca^2+^, ROS burst, activation of MAPK and defence‐related genes, hypersensitive cell death, stomatal closure, and protection against *P. nicotianae* and *Xoo*. Hence, the MOS we reported here can be regarded as a novel DAMP.

In the DAMP‐triggered immunity, the first line leading to active defence responses relies on the perception of DAMPs by PRRs (Zipfel, 2009). It is crucial to study the receptors as well as the recognition process. At present, we do not know how MOS is recognized by plant receptors. Despite the numerous oligosaccharides recognized by plants, only chitin oligosaccharides (CTOS) have been studied thoroughly. The chitin octamer is specific recognized by CERK in *Arabidopsis* or CEBiP in *Oryzae*, but CTOS with low degree of polymerization could inhibit this effect (Liu *et al.*, 2012; Shimizu *et al.*, 2010). It has also been indicated that the sandwich structure of one chitin octamer and two CEBiP receptors was inhibited by deacetylated GlcN‐β‐1,4‐GlcNAc, suggesting that the acetyl groups are essential for the combination of oligosaccharides and receptors (Hayafune *et al.*, 2014). Interestingly, an analogue of CTOS, COS, cannot bind with either CEBiP or CERK (Kaku *et al.*, 2006; Miya *et al.*, 2007). The main difference between COS and CTOS is deacetylation of C‐2 (Yin *et al.*, 2016). These results indicate that in spite of the relatively simple structure of oligosaccharide, a different receptor is responsible for its recognition. Therefore, we infer that there might be a novel receptor for MOS recognition in plants due to its different monosaccharide composition, non‐acetylation, and different DP.

The intracellular Ca^2+^ and apoplastic ROS regulate many processes after the perception of DAMPs, interconnecting branch pathways that amplify and specify the physiological response. They are generally defined as early signalling events during the DAMP‐triggered defence (Garcia‐Brugger *et al.*, 2006). Aziz reported that cellodextrin, OGA, and β‐1,3‐glucan treatment can significantly increase the Ca^2+^ concentration of grape cells, and differs in peak time and intensities (Aziz *et al.*, 2007). It was further confirmed that intracellular Ca^2+^ in tomato guard cells is also involved in oligogalacturonic acid induced stomatal closure (Lee *et al.*, 1999). In addition, Ca^2+^ influx is necessary for ROS production after elicitation (Garcia‐Brugger *et al.*, 2006). ROS, including NO and H_2_O_2_, exhibit both signal transduction and antimicrobial effects in numerous studies resulting in stomatal closure, cell death, phytoalexin production, lipid peroxidation, and defence‐related gene expression, etc. (Gechev and Hille, 2005). In the present study, we confirmed that MOS with a DP of 2–6 stimulated the production of Ca^2+^, ROS, and NO in tobacco guard cells, and also caused an H_2_O_2_ accumulation in both tobacco and rice leaves that was consistent with other reported oligosaccharide elicitors. Moreover, these events occurred within the first minutes to a few hours after MOS perception, suggesting that these rapid reactions can be the characteristic of MOS recognition and development of plant defence responses.

Another early event we investigated was MAPK cascade activation in response to MOS treatment. In plants, the MAPK cascade is required for signal transduction from receptors to downstream components in DAMP‐triggered immunity (Pitzschke *et al.*, 2009). Likewise, MAPK is involved in signalling the intermediate and late defence responses, including the activation of a diverse array of defence genes, cell wall strengthening, phytoalexin biosynthesis, hypersensitive response (HR), and eventually induced resistance (Meng and Zhang, 2013). In tobacco, the MEK2‐SIPK/NTF4 cascade controlled the NOA1‐mediated NO burst, whereas MAPK cascades MEK2‐SIPK/NTF4 and MEK1‐NTF6 regulated the NADPH oxidase‐dependent oxidative burst (Asai *et al.*, 2008). Our transcriptional analysis of MAPK genes showed that the expression level of *NTF6* other than *MEK2* and *WIPK* in tobacco was induced in response to MOS treatment, suggesting that MOS might enhance tobacco resistance through MEK1‐NTF6‐dependent MAPK cascade. Moreover, several rice MAPKs were predicted to play roles in plant immune responses (Reyna and Yang, 2006). Among them, *MPK6* was essential for the chitin‐induced biosynthesis of phytoalexins in rice and was involved in the MAPK cascades of both PTI and ETI (Kishi‐Kaboshi *et al.*, 2010). *MAPK12* was confirmed to positively regulate disease resistance against *Xoo* (Seo *et al.*, 2011). The up‐regulation of *MPK6* and *MAPK12* in response to MOS was consistent with the improved disease resistance. Overall, the MOS perception was sufficient to activate the MAPK signalling in both tobacco and rice.

The subsequent defence responses in plants, such as the hypersensitive response, stomatal closure, pathogenesis‐related gene expression, cell wall stabilization, accumulation of secondary metabolites etc. (Lee *et al.*, 1999; Yin *et al.*, 2010), directly protect plants from pathogen infection. In common with other reported elicitors, local MOS challenges induced tobacco stomatal closure, which was consistent with the ROS change of guard cells mentioned before. In situ MOS treatment on tobacco and rice leaves caused both hypersensitive cell death, which was confirmed by trypan blue staining, and *HSR203J* transcriptional level analysis, although these differed in necrosis phenotype. It should be noted that HR is typically considered a phenomenon from disease resistance (R) gene‐mediated resistance, and is not commonly observed from a MAMP/PAMP/DAMP response (Deslandes and Rivas, 2012; Jones and Dangl, 2006). As far as we know, in all reported oligosaccharides a similar result was only found in COS‐triggered defence responses (Ning *et al.*, 2004; Vander *et al.*, 1998). The stomatal closure as well as HR cell death proved that MOS treatment is able to confine pathogens in local areas. On the other hand, we also adopted the spatial‐time isolation pattern to confirm that MOS could induce systematic resistance for the entire plant. The up‐regulation of defence‐related genes and the accumulation of phytoalexins together confirmed that MOS can not only cause local defences, but also induce systematic resistance against pathogens. Meanwhile, it should be mentioned that *PR‐1a* and *LOX* were both up‐regulated after MOS treatment, suggesting that MOS may simultaneously activate SA‐ and JA‐dependent defence signalling transduction pathways. Taken together, our studies showed that the activation of plant defences using MOS elicitors is probably a valuable strategy for controlling plant disease.

Microbiologists have spend years studying the mechanism of plant growth promoting rhizobacteria (PGPR)‐induced plant resistance. There is relatively little information on the bacterial determinants that trigger induced systemic resistance. However, these determinants are directly recognized by plants as elicitors. In some other cases, an altered functional state of a host molecule (such as a conformational or phosphorylation change), a mislocalized host molecule, the absence of a host molecule, and host molecules liberated by microbe enzymes (such as cell wall fragments of plant origin) could also contribute to plant resistance (Mackey and Mcfall, 2006). These unintended by‐products should also be taken into consideration as eliciting factors or determinants during the plant–PGPR interaction. From this perspective, the numerous oligosaccharides, as well as the degrading enzymes, are supposed to re‐examine their roles in PGPR‐induced systemic resistance.

## Experimental Procedures

### Bacterial strains, plant material, and growth conditions

*Xanthomonas oryzae* spp. *oryzae* PXO99A was grown in nutrition agar medium at 28 °C for 48 h. Cells were then collected by centrifugation and resuspended in distilled water with a final concentration of 10^8^ colony‐forming units (CFU)/mL. *Phytophthora parasitica* var. *nicotianae *strain 025 was kindly provided by Professor Dou from Nanjing Agricultural University. The strain was maintained on Lima Bean Agar (LBA) medium (60 g of lima bean, 20 g of agar, 1, 000 mL of H_2_O) at 25 °C for 5 days before use.

*N. benthamiana* seeds were surface sterilized with 5% (w/v) sodium hypochlorite for 5 min, washed three times with sterilized water and spread out on Murashige–Skoog medium. After 10 days, *N. benthamiana* were then transferred into soil and incubated in a growth chamber with a 16/8 h light/dark cycle at 25 °C.

*O. sativa* seeds were surface sterilized with 70% (w/v) ethanol for 1 min, disinfected with 5% (w/v) sodium hypochlorite for 15 min, and washed three times with sterilized water. After surface sterilization, *O. sativa* were transferred into soil and incubated in a growth chamber with a 16/8 h light/dark cycle at 25 °C.

### Protein expression, purification, and quantification

*E. coli* BL21 harbouring the recombinant Bpman5 gene was inoculated into LB broth containing 50 μg/mL of kanamycin and grown at 37 °C until the OD_600_ reached 0.6 (∼70 min). Then isopropylthio‐β‐galactoside (IPTG) was added with a final concentration of 0.2 mM, and the culture was shaken (200 rpm) at 28 °C for 4–6 h. The cells were harvested by centrifugation and then sonicated on ice to collect soluble proteins. Immobilized metal affinity chromatography and ultrafiltration were adopted for protein purification as described previously (Zang *et al.*, 2015). Protein quantification was carried out using the Bradford method with bovine serum albumin as the standard.

### MOS preparation, quantification, and preliminary purification

The mannan endo‐1,4‐β‐mannosidase BpMan5 was expressed and purified according to our previous study (Zang *et al.*, 2015). Purified BpMan5 (10 U/mL) was incubated with 10 mg/mL LBG at 50 °C for 24 h. Hydrolysis products were collected by centrifugation and filtered using 0.22 μm and 0.45 μm filter membranes. The filtering solution was then processed through 10 and 3 kDa Millipore ultrafiltration devices.

### Hypersensitive response detection

Four‐week‐old *N. benthamiana* leaves and 7‐day‐old rice leaves were inoculated with 200 μg/mL MOS, 500 μg/mL HrpZ_S1_, 500 μg/mL chitosan, 13 μg/mL mannose or sterile water, and necrotic symptoms were recorded within 1–3 days. In addition, the inoculated leaves were soaking in trypan blue dye for 24 h, then cleared with 2.5 g/mL chloral hydrate at least three times. The injection sites were cut off and observed under the microscope.

### H_2_O_2_ assay

Leaves were infiltrated with 200 μg/mL MOS, 500 μg/mL HrpZ_S1_, 500 μg/mL chitosan, 13 μg/mL mannose or sterile water for 6 h, collected, and soaked in PBS buffer containing 0.5% (w/v) DAB for 10 h at 25 °C. Then they were boiled in 95% ethanol for 15 min and the brown precipitates observed.

### Stomatal aperture measurement

*N. benthamiana* leaf epidermis was soaked in MES buffer under light for 3 h to open stomata, and then treated with 200 μg/mL MOS, 500 μg/mL HrpZ_S1_, 1000 μg/mL chitosan, 13 μg/mL mannose or sterile water for 3 h. Stomatal aperture images were captured with an Olympus BX43 microscope (Olympus, Tokyo, Japan) using cellSens Standard Software, and the diameters were measured from 50 randomly selected stomata. Each assay was repeated three times.

### Measurement of Ca^2+^, NO, and ROS in guard cells

*N. benthamiana* leaf epidermis strips were soaked in MES buffer under light for 3 h to open stomata, and soaked in 20 μM Fluo‐3AM, DAF‐2DA or rhodamine 123 at 4 °C for 2 h in darkness. The epidermis strips were washed three time with MES buffer to remove the fluorescent dye and kept at room temperature for 1 h. Finally, the strips were treated with 200 μg/mL MOS, 500 μg/mL HrpZ_S1_, 1000 μg/mL chitosan, 13 μg/mL mannose or sterile water and observed under a Zeiss laser confocal microscope LSM710 (Carl Zeiss, Oberkochen, Germany). Fluo‐3AM, DAF‐2DA, and rhodamine123 were used to analyse Ca^2+^, NO, and ROS accumulation in guard cells, respectively. Each treatment investigated at least three epidermis strips and the experiment was repeated three times.

### Luminol‐based assay for detection of ROS burst in tobacco leaves

The ROS burst assay was performed as described previously with little modification (Bellincampi *et al.*, 2000; Gigli‐Bisceglia *et al.*, 2015). Briefly, tobacco leaf discs (4 mm diameter) of at least four 3–4‐week‐old plants were sampled using a cork borer and floated overnight on sterile water. The following day, one leaf disc per well was placed gently in a 96‐well luminometer plate in which each well contained 200 µl SDW. Luminol/peroxidase working solution (50 µL) was prepared for each well, containing 1% luminol stock solution (15 mg of luminol in 1 mL of DMSO) and 1% horseradish peroxidase stock solution (10 mg of peroxidase in 1 mL of water). The 200 µL of water was gently and rapidly replaced with an equal volume of MOS‐containing solution in each well containing leaf disc. The leaf discs were immediately vacuum infiltrated with the MOS solution, mannose or SDW for 2 min using a desiccator connected to a vacuum pump. Then 50 µL of the luminol/peroxidase working solution was injected into each well and the resulting samples immediately plated in a Cytation 5 Multi‐Mode Reader (BioTek, Winooski, VT, USA) to detect luminescence for 45 min. Each treatment contained four discs and the experiment was repeated three times.

### Induction of systemic resistance

The roots of 1‐month‐old rice were inoculated with 200 μg/mL MOS, 100 μg/mL MOS, 50 μg/mL MOS, 13 μg/mL mannose or sterile water. 24 h later, plants were challenge‐inoculated with *Xanthomonas oryzae* pv. *oryzae* (*Xoo*) PXO99A by the leaf clipping method (Kauffman *et al.*, 1973). After 14 days, the lesion lengths were measured. Six plants were inoculated with *Xoo* strain in each treatment and the experiment was repeated three times. The rice disease was also evaluated by analysis of bacterial growth based on a count of the colony‐forming units as described previously (Ke *et al.*, 2017; Yuan *et al.*, 2016). To measure bacterial growth, approximately 50 mg of *Xoo*‐infected leaves from each plant was examined as one replicate, and a total of three plants for each sample were analysed.

The right‐hand side of 6‐week‐old *N. benthamiana* leaves were infiltrated with 200 μg/mL MOS, 500 μg/mL HrpZ_S1_, 1000 μg/mL chitosan, 13 μg/mL mannose or sterile water for 4 h, and left‐hand sides of *N*. *benthamiana* leaves were inoculated with a 7 × 7 mm hyphal plug of *P. parasitica* 025 at 25 °C in darkness. Disease symptoms were measured after 48 h, and then decolorized in ethanol. Inhibition rate (%) = (average diameter of control‐average diameter of treatment)/average diameter of control × 100%. Each treatment contained six plants and the experiment was repeated three times.

### RNA isolation and real‐time PCR

For NO/ROS/MAPK analysis, the leaves were cut off 3 h after foliage application of 200 mg/L MOS. For HR analysis, plants were foliage application of 200 mg/L MOS for 6 h. For defence‐related gene expression, the upper leaves of tobacco seedlings were collected 12 h after foliage application of 200 mg/L MOS on lower leaves, whereas rice leaves were cut off 12 h after root irrigation with 200 mg/L MOS. Total RNA was extracted using a plant RNA kit (Omega Bio‐Tek, Norcross, GA, USA) according to the manufacturer’s instructions. First‐strand cDNA was synthesized using reverse transcriptase (Takara Bio Inc., Dalian, China) with oligo dT primers. Real‐time PCR was performed using SYBR Premix Ex Taq polymerase (Takara Bio Inc.) on either an ABI 7300 Fast Real‐time PCR System (Applied Biosystems, Foster City, CA, USA) or a CFX96 connect Real‐time PCR System (Bio‐Rad, Hercules, CA, USA). The *EF‐1α* gene was used as an internal reference. At least three replicates were carried out for one sample. The experiments were repeated three times.

### Measurement of four phytoalexins variation in rice seedling leaves

The four phytoalexins (momilactone A, phytocassane A, phytocassane D, and phytocassane E) in rice were detected using LC‐MS according to Xie (Xie *et al.*, 2017). Briefly, 10‐day‐old rice seedlings were root‐irrigated with MOS and the rice leaves were then collected after 12, 24, 48, and 60 h. Accurate 100 mg samples of rice leaf tissue were incubated with 80% methyl alcohol for 48 h. Four‐layer gauze was used to filter the solution and the leaf residue was discarded. Crude extracts were evaporated to dryness and dissolved in 1 mL mixed liquor (ethanol:water:acetonitrile:acetic acid = 79:13.99:7:0.01, v/v/v/v), with 5 μL of this solution used for LC‐MS analysis. The LC‐MS experimental conditions were mobile phase A, 0.02% methane acid; mobile phase B, acetonitrile; flow rate, 0.2 mL/min. According to phytoalexin and precursor structures in rice, multiple reaction monitoring patterns were used to conduct relative quantitative analyses for rice phytoalexin. According to the method of Shimizu (Shimizu *et al.*, 2008) the parent ion/daughter ion of momilactone A was 315/271 and the parent ion/daughter ions of phytocassane A, phytocassane D, and phytocassane E were all 317/147.

### Statistical analysis

Three independent experiments were performed for each assay. The data were analysed by Fisher’s least‐significant difference test (*P* < 0.05) with SPSS software.

## Competing interests

The authors declare no conflict of interest.

## Supporting information


**Fig. S1** Expression and purification of BpMan5.

**Fig. S2** Original image of intracellular Ca^2+^ in guard cells of *N. benthamiana*.

**Fig. S3** Original image of NO (A) and ROS (B) generation in guard cells of *N. benthamiana.*

**Fig. S4** Original image of the stoma aperture in tobacco.

**Table S1** Quantification of various kinds of oligosaccharides in a hydrolysis mixture.

**Table S2** Primers designed for real time PCR experiment.
