# Supplementary figures and images for: Mannan oligosaccharides trigger multiple defence responses in rice and tobacco as a novel danger‐associated molecular pattern
Source: Mol Plant Pathol. 2019 May 16;20(8):1067–79. doi: 10.1111/mpp.12811 (PMC6640537; doi:10.1111/mpp.12811)

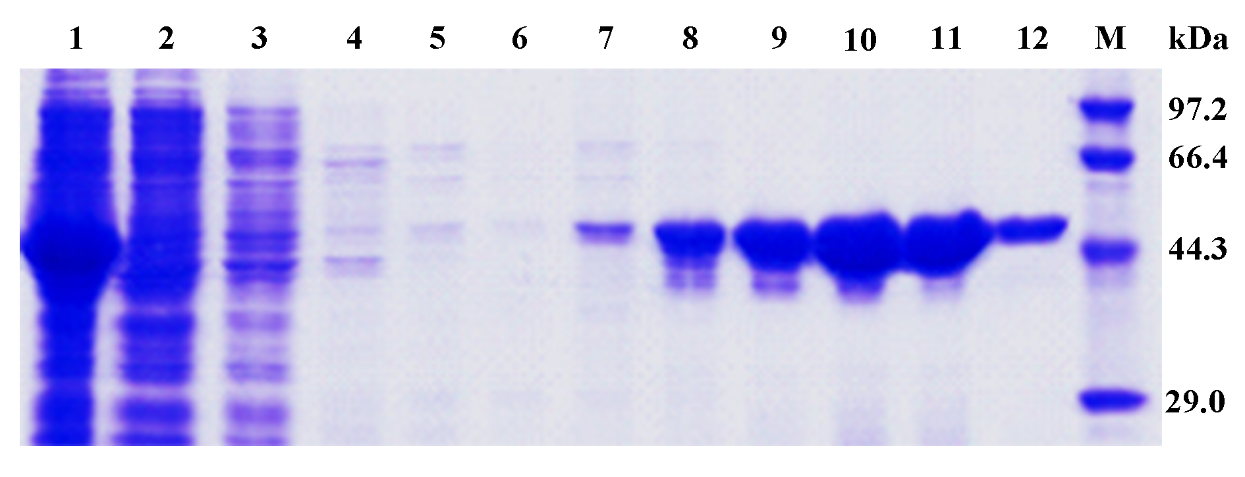


**Fig S1 Expression and purification of BpMan5.**

Supplement: Supplementary file 1 — Fig. S1 Expression and purification of BpMan5. [file MPP-20-1067-s001.docx]

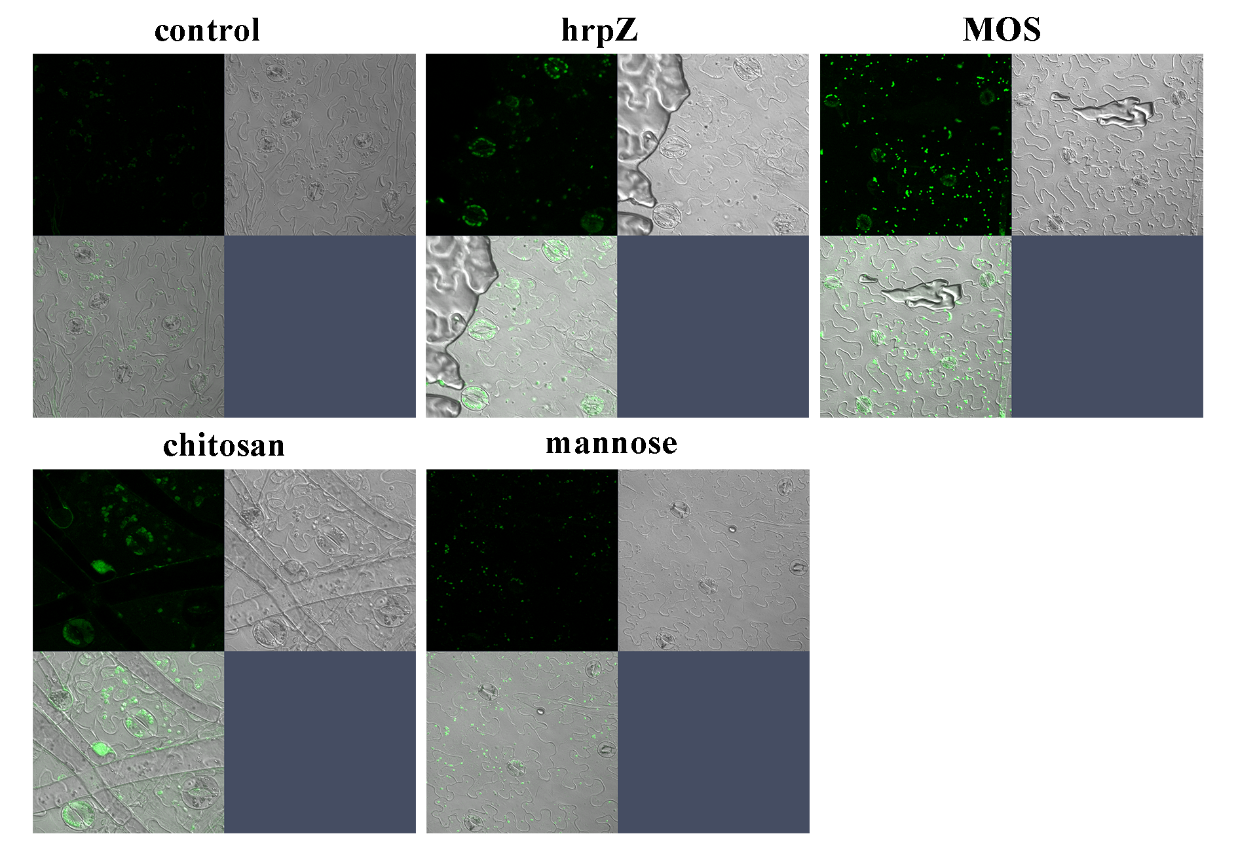


**Fig S2 Original image of intracellular Ca2+ in guard cells of *Nicotiana benthamiana*.**

Supplement: Supplementary file 2 — Fig. S2 Original image of intracellular Ca2+ in guard cells of N. benthamiana. [file MPP-20-1067-s002.docx]

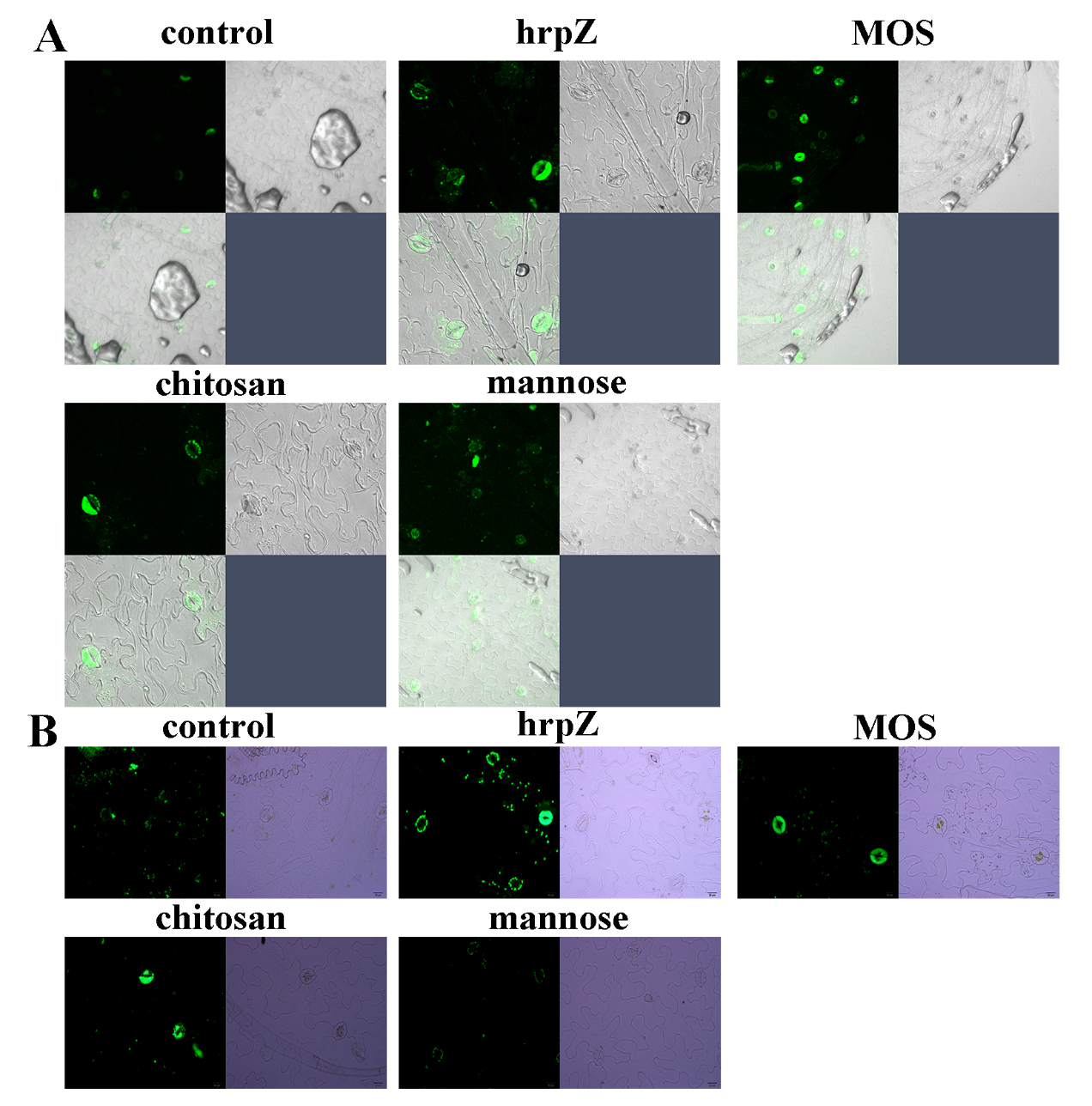


**Fig S3 Original image of NO (A) and ROS (B) generation in in guard cells of *Nicotiana benthamiana*.**

Supplement: Supplementary file 3 — Fig. S3 Original image of NO (A) and ROS (B) generation in guard cells of N. benthamiana. [file MPP-20-1067-s003.docx]

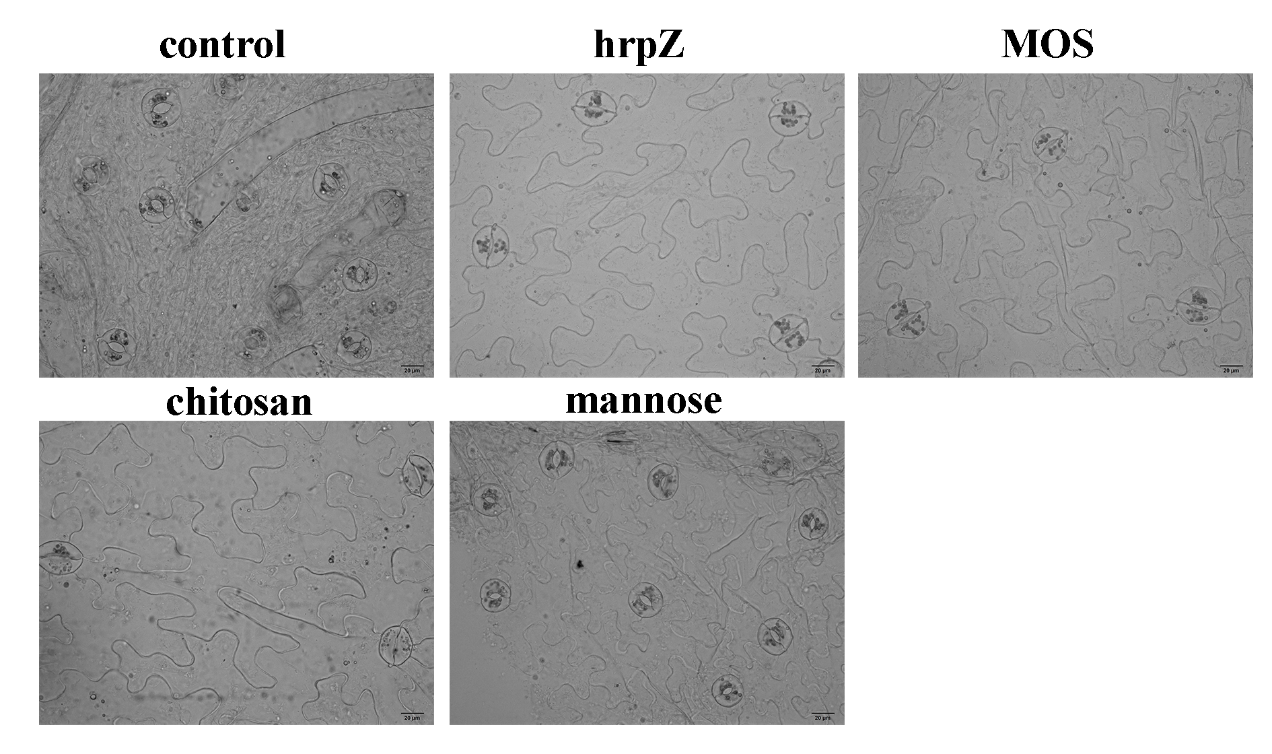


**Fig S4 Original image of stoma aperture in tobacco.**

Supplement: Supplementary file 4 — Fig. S4 Original image of the stoma aperture in tobacco. [file MPP-20-1067-s004.docx]
