# Supplementary material for: Mannan oligosaccharides trigger multiple defence responses in rice and tobacco as a novel danger‐associated molecular pattern
Source: Mol Plant Pathol. 2019 May 16;20(8):1067–79. doi: 10.1111/mpp.12811 (PMC6640537; doi:10.1111/mpp.12811)
Supplement: Supplementary file 5 — Table S1 Quantification of various kinds of oligosaccharides in a hydrolysis mixture. [file MPP-20-1067-s005.docx]

**Table S1 Quantification of various kinds of oligosaccharides in hydrolysis mixture.**

| **composition** | **M1** | **M2** | **M3** | **M4** | **M5** | **M6** |
| --- | --- | --- | --- | --- | --- | --- |
| **Concentration （mg/ml）** | 0.07 | 0.27 | 0.22 | nd | 0.58 | 0.04 |

M1: mannose, M2: mannobiose, M3: mannotriose, M4: mannotetrose, M5: mannopentaose, M6: mannohexaose.
