# Supplementary material for: Mannan oligosaccharides trigger multiple defence responses in rice and tobacco as a novel danger‐associated molecular pattern
Source: Mol Plant Pathol. 2019 May 16;20(8):1067–79. doi: 10.1111/mpp.12811 (PMC6640537; doi:10.1111/mpp.12811)
Supplement: Supplementary file 6 — Table S2 Primers designed for real time PCR experiment. [file MPP-20-1067-s006.docx]

**Table S2 Primers designed for real-time PCR experiment.**

| **Primer name** | **Oligonucleotide sequence (5'-3')** |
| --- | --- |
| NbEF1α-F | ATGATTACTGGTACCTCCCG |
| NbEF1α-R | ACCTAGCCTTGGAATACTTG |
| NbPR1a-F | CGTTGAGATGTGGGTCAATG |
| NbPR1a-R | CCTAGCACATCCAACACGAA |
| NbrobhA-F | GAAGGCGGAGTTAAGGAGAT |
| NbrobhA-R | GAGCTCTATGAGCGCTGGAA |
| NbrobhB-F | GTGATGCTCGTTCTGCTCTT |
| NbrobhB-R | CTTTAGCCTCAGGGTGGTTG |
| NbNIA1-F | CATTCCTCGACGTGAAAGGT |
| NbNIA1-R | AGAATTGCCTGCATGACTTG |
| NbNIA2-F | GTGTGGCCCTAATTCCAAGA |
| NbNIA2-R | CGTCAATAACGGCACAGAGA |
| NbERF1-F | GCTCTTAACGTCGGATGGTC |
| NbERF1-R | AGCCAAACCCTAGCTCCATT |
| NbLOX-F | CCTTAAGAGGAGATGGAACT |
| NbLOX-R | TCTAAGCTCATAAGCAATGG |
| NbHSR203J-F | GAGCCCTGGCTCAACAATTA |
| NbHSR203J-R | CTCCGATTTGCTCCGATAAG |
| NbMEK2-F | ACTTCTCCGAACTTGAGCGTAT |
| NbMEK2-R | CCTCGTGGTTACCGTAGATGACT |
| NbNTF6-F | TCCCTCCTATTCAACCTGTCG |
| NbNTF6-R | TTGCTACCTCCTCCTTCGTCT |
| WIPK-F | TAACTCACGGCGGACAATA |
| WIPK-R | TCCATAAGCACCACGACCA |
| OsEF1α-F | TTTTCGTTTGTGCTATTCC |
| OsEF1α-R | AAATCCATTAGGCTTTCAG |
| OsPR1a-F | CGCAGCAACCAACCAATC |
| OsPR1a-R | GTAGGAAAAAAGGTAAAAAAGCAAA |
| OsERF1-F | CTGAAGCACGCAGGATT |
| OsERF1-R | TTAGCAGATGGGTAGACAAA |
| OsLOX-F | GGCTCATCGGCGTCTTG |
| OsLOX-R | CCTCCTTGTTCTCCTCCACC |
| OsHSR203J-F | TTGGCCTGAGCTTGACCTT |
| OsHSR203J-R | CCACCCTTTATCATTGGACACT |
| OsMAPK12-F | TTGCCCGCACTACAGTAAGCC |
| OsMAPK12-R | GACAATCCATCGACCGACCCT |
| OsMEK2-F | ACGAGATACATTTACTGGGACA |
| OsMEK2-R | CAATTCTAGCATAACGAGGC |
| OsMPK6-F | AGCATTGTCAGAGGAGCACT |
| OsMPK6-R | TCTGAGGTGGTACGAGCAAG |
